# Delays in the Autism Spectrum Disorder Diagnostic Pathway: An Audit of Wait Times for Initial Assessment and Post-Diagnosis Support in Greater Preston

**DOI:** 10.1192/bjo.2025.10691

**Published:** 2025-06-20

**Authors:** Cydney Tripley, Maria Hall

**Affiliations:** Manchester University, Manchester, United Kingdom

## Abstract

**Aims:** Timely initiation of assessment for Autism Spectrum Disorder (ASD) is crucial as delays can significantly impact development of children and family well-being. This audit aimed to assess the adherence to National Institute for Health and Care Excellence (NICE) guideline recommendations of timely initiation of ASD assessment and follow-up care in the Greater Preston Area.

**Methods:** A retrospective observational study was conducted, using electronic patient records of patients referred to the ASD diagnostic pathway and listed for further investigation in 2022 in the Greater Preston area. Data on wait times between referral and first appointment and times between diagnosis and follow-up appointment were collected and analysed. Patients eligible for this study were under 18 years of age, living in the Preston area and had been referred to the ASD diagnostic pathway and listed for further investigation in 2022. 37 school-age and 48 preschool-age children were included in this study.

**Results:** It was found that 18.9% of school-age and 16.7% of preschool-age children were seen within the 13-week window between referral and first appointment recommended by NICE guidelines. This study also showed that 18.9% of school-age children and 20.8% of preschool-age children received a follow-up appointment within the 6-week guideline.

**Conclusion:** This study found significant delays in accessing the ASD diagnostic pathway and follow-up care which indicates the Greater Preston area is not in adherence with NICE guidelines. Changes are necessary to address these gaps and ensure timely support for children affected by ASD.

